# Quantitative Bio-Imaging Tools to Dissect the Interplay of Membrane and Cytoskeletal Actin Dynamics in Immune Cells

**DOI:** 10.3389/fimmu.2020.612542

**Published:** 2021-01-11

**Authors:** Falk Schneider, Huw Colin-York, Marco Fritzsche

**Affiliations:** ^1^ Medical Research Council (MRC) Human Immunology Unit, Weatherall Institute of Molecular Medicine, University of Oxford, Oxford, United Kingdom; ^2^ Kennedy Institute for Rheumatology, University of Oxford, Oxford, United Kingdom; ^3^ Rosalind Franklin Institute, Harwell Campus, Didcot, United Kingdom

**Keywords:** plasma membrane, actin cytoskeleton, fluorescence correlation spectroscopy, fluorescence recovery after photobleaching, immune cells, metal induced energy transfer, volumetric imaging, quantitative imaging

## Abstract

Cellular function is reliant on the dynamic interplay between the plasma membrane and the actin cytoskeleton. This critical relationship is of particular importance in immune cells, where both the cytoskeleton and the plasma membrane work in concert to organize and potentiate immune signaling events. Despite their importance, there remains a critical gap in understanding how these respective dynamics are coupled, and how this coupling in turn may influence immune cell function from the bottom up. In this review, we highlight recent optical technologies that could provide strategies to investigate the simultaneous dynamics of both the cytoskeleton and membrane as well as their interplay, focusing on current and future applications in immune cells. We provide a guide of the spatio-temporal scale of each technique as well as highlighting novel probes and labels that have the potential to provide insights into membrane and cytoskeletal dynamics. The quantitative biophysical tools presented here provide a new and exciting route to uncover the relationship between plasma membrane and cytoskeletal dynamics that underlies immune cell function.

## Introduction

Life is dynamic. Cellular components are in constant motion bridging various time- and length-scales. This includes the plasma membrane and the cortical actin cytoskeleton, which form a dynamic interface between the cell and its environment, working together to control cellular signaling and morphology as well as to maintain the mechanical integrity of the cell. It is becoming increasingly clear that the dynamics of the cortical actin cytoskeleton and the plasma membrane are intimately linked to immune cell function, playing a critical role in, for instance, the regulation of receptor organization, granule section, and specific cytoskeletal protrusions (1–4). Despite their individual importance, how both the membrane and the cortical actin cytoskeleton dynamics are coupled, and how feedback between the two structures shapes their interplay in immune cells remains unknown. Crucially, to understand the functional significance of this interplay, measurement techniques are required that allow the dynamics of actin and membrane to be captured simultaneously, enabling their direct correlation in both space and time.

### Actin-Membrane Interactions at a Glance

**Figure 1:**
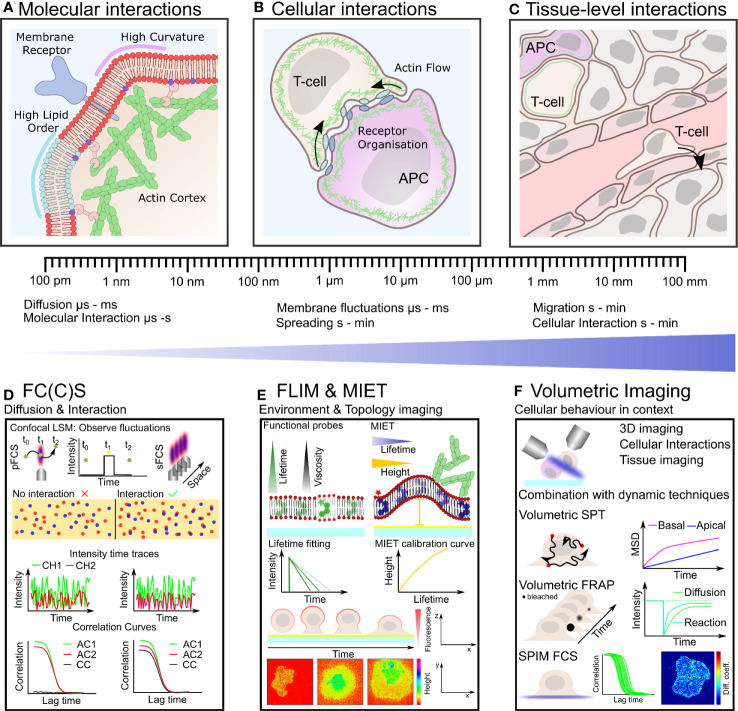
Plasma membrane organization and actin cytoskeletal dynamics are intrinsically linked to immune cell organization and function over many length- and time-scales **(A–C)**. Techniques with the ability to quantify simultaneous dynamics present an exciting route to understand the interplay of cortical actin and plasma membrane in immune cells **(D–F)**. **(A)** On the nano-scale actin filaments are turning over and binding the plasma membrane influencing its organization which in turn impacts on actin organization and the distribution of receptors, lipids and other membrane constituents. **(B)** Defined actin and membrane flows, organization and their integrity are crucial for cell-cell contacts such as during immunological synapse formation on the meso-scale. **(C)** Within tissue, immune cells must navigate through biophysically diverse environments, relying on both a dynamic plasma membrane and actin cytoskeleton to carry out their function. **(D)** Fluorescence correlation spectroscopy (FCS) and FCCS measure fluctuation of fluorescently labeled molecules diffusing through the focus of a confocal microscope. Typical transit times range from µs to hundreds of ms highlighting the large dynamic range and unrivaled temporal resolution of these techniques. Auto-correlation (AC) of the intensity traces (in two spectral channels CH1 and CH2) allows to calculate diffusion coefficients and interactions (cross-correlation, CC). **(E)** Fluorescence lifetime imaging (FLIM) allows to monitor changes in fluorescence lifetime. Acquiring FLIM images typically takes seconds to minutes. Functional probes can change their lifetime in accordance to their environment sensing, for example, viscosity. In metal induced energy transfer (MIET) the lifetime change is correlate with the distance to the surface and allows the recalculation of heights yielding the membrane topology, for example, during cell spreading. **(F)** Volumetric imaging using, for instance, a light-sheet approach allows to image the cellular context in 3D at moderate time resolution (down to seconds). Combination with the dynamics techniques, single particle tracking (SPT), fluorescence recovery after photobleaching (FRAP), and FC(C)S represents a promising route for mapping plasma membrane and actin dynamics in the full physiological setting by correlating their time- and length-scales.

The interactions between the membrane and the actin cortex are numerous and complex and have been the subject of intense research (5, 6) (**Figure 1A**). Underlying the plasma membrane the actin cortex exists as a densely cross-linked meshwork of filamentous actin (F-actin) formed by the polymerization of globular actin (G-actin) monomers undergoing constant turnover on the second time-scale (7). The dynamic architecture of the actin cortex is governed by two primary modes of F-actin polymerization driven by either Arp2/3 or formin nucleation leading to constantly varying actin mesh-sizes from tens of nanometers to microns (8). The dynamic nature of the cortex as well as its mechanical plasticity is largely mediated by a variety of myosin motors that cross-link individual filaments and induce active mechanical stress within the network (8).

Biochemically linking the actin cortex and the plasma membrane are a series of specific protein-protein and protein-lipid interactions (9). One of the most important membrane components mediating this interaction is the glycolipid phosphatidylinositol-bisphosphate, PIP2. By binding the ERM (ezrin, radixin, and moesin) proteins, an actin binding family of proteins, PIP2 provides a linkage between cytoskeleton and membrane (**Figure 1A**). In addition to the ERM proteins, WASP and WAVE, key regulators of Arp2/3 driven actin polymerization, are also able to bind PIP2 in the membrane, leading to active polymerization of F-actin at the plasma membrane (6). Notably, the conserved diversity of specific linkages between cortex and membrane is indicative of the importance of the tight coupling of these structures for their function within the cell.

In addition to the specific molecular interactions between the plasma membrane and actin cortex, there are a number of more general biophysical interactions that have been well characterized *in vitro* (10), for example, the local charge of the membrane has been shown to influence actin binding (11). Furthermore, curvature introduced by the polymerization of the actin cytoskeleton during the formation of specific protrusions can influence the diffusion and distribution of membrane proteins (12, 13). Conversely, membrane curvature induced by the physical membrane microenvironment can lead to actin polymerization (14). Local changes in actin polymerization can also induce changes in the rate and diffusion mode of lipid and protein components as well as the membrane tension (15–18). Crucially, membrane composition, dynamics, and organization influence the underlying actin cytoskeleton. Similarly, the dynamics and architecture of the actin cytoskeleton has been shown to influence the plasma membrane (19). Therefore, the complex interplay between membrane and cortical cytoskeleton makes assessing causal links challenging, highlighting the unmet need for techniques that allow the dynamics of both components to be quantified simultaneously in space and time.

### Actin-Membrane Interactions in Immune Cells

Many stages of the immune response, for example, antigen recognition, rely on the integration of information by immune cells from their environment, often involving the formation of highly specialized cell-cell contacts, such as the immunological synapse (IS) that forms between T-cells, B-cells, and antigen presenting cells (APCs) (**Figure 1B**). At these contacts, immunological signaling is initiated and propagated *via* the interactions of a wide array of molecules, occurring on and in the proximity of the plasma membrane. Owing to this, the dynamics of both the plasma membrane and the underlying actin cytoskeleton have a profound impact on the organization and dynamics of crucial signaling molecules. The process of immune cell activation spans a range of time- and length-scales starting with nano-scale reorganization and receptor engagement (sub-second), actin polymerization driven retrograde flow (seconds), to micron-scale cellular activation, spreading, and cytokine secretion (up to hours) (20–22).

During T-cell activation and IS formation, one of the key steps in the adaptive immune response, there has been increasing interest in the role of specific cytoskeletal protrusions in the initiation and orchestration of early T-cell signaling at the plasma membrane (23–25). Microvilli at the T-cell surface have been shown to provide an efficient means of environment scanning, while association of ERM proteins to the plasma membrane interface has been shown to lead to the accumulation of signaling molecules at microvilli (23, 24, 26). Following T-cell receptor (TCR) triggering, the T-cell undergoes a dramatic morphological change driven by the rapid polymerization of F-actin. This results in the formation of an increased contact area between the two cells, which is characterized by the retrograde flow of actin filaments within a lamellipodial structure at its periphery as well as a ramified actin network at its center (27). Crucially, TCRs are trafficked in coordination with the F-actin flow toward the center of the IS and the continued flow of actin has been shown to be necessary for continued activation, sustaining PLCγ1 signaling (28–30). Notably, once the IS has formed, recent evidence suggests that tension generated by specific dynamic actin structures influence the symmetry and lifetime of the IS (31).

In contrast to its polymerization, the depletion of actin has also been shown to play a role in immune cell function. Targeted killing by cytotoxic T lymphocytes requires the precise spatio-temporal control of actin depletion with recent studies pointing to a complex and intricate mechanism, whereby the density of the cortical actin underlying the membrane is tuned by the interaction of the kinase PIP5K and the loss of the charged PIP2 lipid (32, 33).

Like the cortical actin cytoskeleton the plasma membrane is a dynamic structure, constantly in motion and continuously reorganizing (34, 35). The presence of defined domains or rafts (tens of nanometer in diameter) has been evoked to explain a number of phenomena, including lateral heterogeneity of the plasma membrane and, for example, the non-random distribution of membrane proteins on the cell surface (35–38). Along these lines, the reorganization of immune receptors by specific incorporation into such structures has been described (39–41). This is based on a biophysical property of the membrane and the proteins themselves: proteins can exhibit a preference for different lipid environments preferring, for instance, a densely packed lipid environment (liquid ordered phase, highly viscous) enriched in saturated lipids and cholesterol (42, 43). In contrast, other proteins can prefer the liquid disordered membrane environment, rather associating with loosely packed, unsaturated lipids resulting in a low viscosity environment. This could act as means of increasing interaction likelihood and forming signaling platforms (44, 45). The degree of order and the viscosity of the membrane are primarily tuned by the cholesterol content and in addition likely by its specific interactions with proteins and lipids (46–48). Intriguingly, the attachment of actin filaments to the membrane has been seen to influence membrane organization, resulting in the formation of ordered domains (49, 50), providing a potential mechanism for actin to indirectly influence membrane protein organization.

As is evident, studies of the dynamics of the cytoskeleton and membrane have led to important insights into the function of immune cells (51–53). Despite this, there are only a limited number of studies that have attempted to address the correlated biophysical and biochemical dynamic mechanisms whereby membrane and actin work together in immune cells. Recent advances in correlative imaging such as the combination of super-resolution microscopy and electron microscopy have allowed for detailed insights into structural links between plasma membrane and cortical actin organization (54–56). However, these approaches only allow snapshots of a constantly evolving structure and thus do not allow the dynamic interplay to be followed live, and therefore make assessing causality challenging. Furthermore, little is known about how these interactions influence the behavior of cells in more complex tissue environments or in their full physiological setting, with our knowledge often restricted to *in vitro* single cell studies (**Figure 1C**). This has primarily been due to a lack of accessible technologies with sufficient temporal (< ms binding and transport events) and spatial resolution (single proteins on the order of nanometres can effect changes on cellular level beyond tenth of micrometers) to assess the correlated dynamics of the plasma membrane and the actin cytoskeleton without perturbing the system. In addition to this, such lack of technology has been confounded by a lack of membrane and actin probes that can operate at physiological conditions and offer reliable performance within the cellular environment.

Here, we review recent advances in both dynamic measurement techniques and actin/membrane probes that have not yet been widely applied to study immune cells. In our view, these methods present a significant opportunity to address the complex interplay between these two systems crucial to the immune response by simultaneous quantification of both membrane and actin dynamics.

## From Fluorescence Imaging to Quantification of Dynamics

Live-cell fluorescence imaging is the method of choice to understand the behavior of dynamic biological processes owing to the specificity of labeling structures of interest and the minimal invasiveness of the approach. Observing events crucial to the immune response occurring in live cells has long been performed by employing fluorescence microscopy with confocal and total internal reflection fluorescence (TIRF) time-lapse imaging due to their optical sectioning capabilities yielding key insights into the dynamic nature of these processes (57, 58). This approach has revealed, for example, the formation and trafficking of T-cell receptor clusters (21, 59), which is crucial for initiation and continuation of signaling and has been shown to be strongly regulated by the dynamic interplay of the membrane and cortical actin cytoskeleton flows (60, 61). Despite their success, imaging alone restricts the level of quantitative information that can be extracted from the biological system of interest, for instance, due to the limited time resolution of time-lapse imaging acquisitions (tens of ms to s).

An alternative quantitative route to assessing transient processes such as cellular reorganization driven by molecular diffusion, binding-kinetics, or flow has been the use of dynamic techniques such as single particle tracking (SPT), fluorescence recovery after photobleaching (FRAP), or fluorescence fluctuation spectroscopy (FFS) approaches. For a detailed technical description, we refer the reader to (62–64). In immunology, these traditional techniques and their advancements have, for instance, been used to investigate the diffusion properties of key signaling molecules such as BCR, CD1d, TCR, CD45, or Lck in live cells (65–74).

## Fluorescence Fluctuation Based Approaches to Assess Simultaneous Dynamics

The spatial heterogeneity across microns along with fast molecular interactions within the cell membrane represent a challenge to all dynamic techniques which rely on maintaining single molecule sensitivity in the crowded cellular environment. Similar to fluorescence time-lapse imaging FRAP and SPT are conventionally limited to resolving processes in the ms time regime. Using fluctuation based techniques such as fluorescence correlation spectroscopy (FCS) offers unmatched temporal resolution (down to ns) to cover the range from very fast molecular binding dynamics (µs) to motion of large protein complexes in the membrane (hundreds of ms). Unfortunately, the spatial resolution remains diffraction limited (~200 nm). Thus, inherently one will average many molecular interactions missing precise details on dynamics and potential sub 100 nm spatial heterogeneity which could be functionally important, for instance, during receptor-ligand engagement. The combination of stimulated emission depletion (STED) super-resolution microscopy with FCS has proven itself as a valuable remedy and a tool to assess nano-scale diffusion directly at the relevant spatio-temporal scales (75, 76). It has revealed a vast heterogeneity in diffusion behaviors of membrane constituents caused by interactions with lipid domains, transmembrane proteins, or the cortical actin cytoskeleton (16, 17, 77). While STED-FCS offers very high spatial resolution in living specimens (<50 nm), it is limited by comparatively high light exposure, requires dedicated equipment (depletion beam), and necessitates special dyes (75).

By sampling not only a single point, but rather a linear or circular region, scanning fluorescence correlation spectroscopy (sFCS) studies offer not only increased statistical power over conventional FCS but also limited phototoxicity (78, 79), and allow spatial heterogeneities in molecular diffusion dynamics to be accounted for (**Figure 1D**). The increased statistical power of sFCS can be exploited to decipher the diffusion mode of the molecule of interest (determining if a molecule undergoes free Brownian or hindered diffusion (80) due to nano-scale interactions). On the one hand, changes in diffusion behavior can initiate signaling pathways and on the other hand can be indicative of a cellular state such as activation (81, 82). Similar to STED-FCS, the statistical analysis of sFCS data can yield details of dynamic molecular organization but notably does not rely on any special equipment or dyes and can be performed on any turn-key confocal laser scanning microscope (79, 80). Critically, two-color scanning fluorescence cross-correlation spectroscopy (sFCCS) data can be used to characterize the dynamics of two species of interest, exploring their interplay in detail (83–86) (**Figure 1D**). Super-resolved (STED) cross-correlation studies have not yet been achieved, but in combination with beam scanning bare potential to uncover short-lived interactions (87, 88). Together with harnessing the statistical power of scanning approaches, we anticipate that the advances in fast photon-counting acquisitions, high-count rate FCS and thus fast photon filtering may pave the way to the realization of such techniques (89, 90). Cross-correlation studies offer a unique opportunity to quantify cytoskeletal and membrane dynamics simultaneously, for example, probing the dynamic interactions between signaling molecules at the plasma membrane and the flow of the actin cytoskeleton during synapse formation, allowing cross-correlation on short (µs to ms) time-scales. Such correlated, simultaneous acquisitions represent a promising way to dissect causation and may decipher when the actin cytoskeleton is driving the membrane organization and vice versa.

Imaging larger regions allows for the mapping of diffusion across space and delivers increased spatial information at the expense of temporal resolution (> ms). Both TIRF and single-plane illumination (SPIM) schemes have been combined with camera based FCS acquisitions yielding similar and even larger statistics compared to sFCS (91–93). Crucially, these techniques provide subcellular or cellular imaging, contextualizing the dynamic measurements and allowing routine correlation with specific compartments of the cell. Expanding such approaches using image mean squared displacement (iMSD) analysis can even yield insights into the diffusion modes with a statistical power similar to STED-FCS and sFCS (94, 95). For camera based acquisitions the frame time (of about 1 ms minimum) represents the most common bottleneck for resolving fast diffusion (96). Given recent advances in camera technology this will likely not pose a limitation for much longer. For example, interferometric scattering (iSCAT) microscopy, which relies on collecting scattered light from the sample rather than fluorescence emission, allows frame rates of multiple kilo Hertz covering most of the range of dynamic processes in biology (97–99).

## Exploiting Fluorescence Lifetime to Measure Dynamics and Topology

The time a fluorophore spends in the excited state is termed fluorescence lifetime and can be used as an additional means for introducing contrast in fluorescence microscopy with the fluorescence lifetime strongly depending on the environmental conditions and fluorophore properties (62). The unrivaled temporal resolution of fluorescence fluctuation approaches offer a promising route to decipher lateral membrane organization. Yet, biology operates in all three spatial dimensions, for example, actin polymerization causing plasma membrane deformations, and the aforementioned methods are largely blind to changes in axial organization. In the following, we discuss a possible remedy exploiting fluorescence lifetime modulation.

The recent advances in commercially available fluorescence lifetime imaging (FLIM) platforms enable fast acquisitions (few seconds per frame) and easy access to this microscopy modality (100). Typically, fluorescence lifetime information is used as an intrinsic method to generate contrast in unlabeled samples (using auto-fluorescence) or in conjunction with Foerster resonance energy transfer (FRET), where the lifetime shortening of the fluorescence donor is used to calculate the distance between two fluorochromes (donor and acceptor) revealing molecular interactions or conformational changes (101) (**Figure 1E**). A versatile variation of FRET makes use of the lifetime and fluorescence quenching abilities of thin metal films on the glass coverslip. In metal induced energy transfer (MIET), the lifetime can be used to calculate the height (distance from the quenching surface) of a fluorophore with nanometer precision across a range of 0-150 nm (102) (**Figure 1E**). The dynamic range and localisation precision can be tuned by the coating material (most commonly gold and more recently graphene) (103–105). MIET displays a great opportunity to explore membrane topology and curvature (106), for example, in common T cell surface interaction studies using plasma membrane markers, as has been applied to study the epithelial-to-mesenchymal transition of epithelial cells (107). This becomes even more powerful when combined with two-color labeling (108, 109), allowing the simultaneous spatio-temporal quantification of the actin cortex and plasma membrane topology, which may elucidate microvilli structures and allow axial mapping of segregation and IS organization (109). Specifically, it could be used to differentiate actual protein clusters from axially stacked molecules (as in microvilli). MIET can be combined with FCS (or rather its cousin fluorescence lifetime correlation spectroscopy) allowing for height dependant dynamic measurements, giving the opportunity to separate receptor dynamics proximal and distal from the surface within one measurement in a few seconds, allowing key insights into the mobility of receptors in the vicinity of specific actin structures (110). As a further extension, the combination of fluorescence lifetime imaging with functional probes paves the way for some exciting applications, whereby membrane topology can be correlated with other readouts, such as membrane tension, curvature, or order (see below).

## Fast Volumetric Imaging and Its Combination With Dynamic Techniques

The aforementioned approaches give highly detailed insights on fast time- and short length-scales. Nevertheless, the actin cortex plasma membrane interplay also affects larger-scale cellular dynamics such as pushing/pulling of the membrane, cell migration and sampling of the immediate surroundings (see introduction) (**Figure 1C**). The investigation of these processes necessitate tools that are able to operate in in all three dimension (3D), capturing the complex geometries and topologies of cellular and multicellular samples. In biology, 3D (volumetric) imaging has typically been achieved using axial scanning confocal microscopy, and more recently using super-resolution techniques such as 3D-SIM. Unfortunately, such techniques are often restricted to relatively long (several seconds to minutes) scan times, limiting their application to slowly evolving biological systems. To investigate transient processes in a more physiological setting (compared to a planar coverslip), great advances in volumetric and *in vivo* imaging have been made, primarily based on the use of light sheet technologies, allowing for rapid 3D acquisition (111–116) (**Figure 1F**). These volumetric imaging approaches present an opportunity to overcome a long-standing issue for the investigation of plasma membrane and actin cortex interactions in lymphocytes such as T-cells. Typically, due to restrictions imposed by conventional imaging methods, specific activation is achieved by replacing the antigen presenting cell by a coverslip coated with an activing molecule or a supported lipid bilayer presenting target molecules (117, 118). This approach has led to a great number of important insights into immune cell biology, yet it omits a large proportion of the biological complexity and three-dimensional geometry present within the physiological interactions between immune cells and target cells. Consequently, the opportunity to investigating plasma membrane and actin cortex interactions in physiological geometry using volumetric imaging is likely to yield great insights (33, 119, 120).

Crucially, instead of acquiring time-lapse imaging alone, combing the aforementioned dynamic techniques such as FCS, FRAP, or SPT with light-sheet imaging has the great advantage of providing spatial context for the observations (121). Notably, in contrast to FCS, the FRAP method can not only extract the dynamics of one species of interest, but can also quantify reaction processes, such as the binding of actin monomers within the actin cortex (122). The combination of FRAP with volumetric imaging therefore represents an exciting opportunity to correlate diffusive processes, for example, at the plasma membrane with the reaction driven turnover of the actin cortex beneath. Such technologies will likely be key in providing insights into the correlated dynamics of immune cell membrane and actin cytoskeleton within physiologically relevant environments (123).

All these techniques and ideas, of course, rely on appropriate and non-perturbing labeling strategies. Excitingly, a large variety of actin and membrane labels are now available.

## Labels and Probes for Quantification of Plasma Membrane Dynamics

A variety of approaches can be chosen to label the plasma membrane. Broadly speaking, labels can be divided in specific labels, binding to or mimicking a certain lipid, and non-specific labels, displaying usually hydrophobic compounds, which insert into the membrane. The former can be used to study a specific lipid or pathway, the latter as a general membrane label.

Non-specific labels such as DiO or DiI have been around for decades and have even been used for *in vivo* cell tracking (124, 125). They conveniently incorporate quickly into the membrane by incubation alone which works well with model membranes but can require optimisation for live-cell membrane staining (126). Homogeneous membrane labeling can also be achieved using various other commercial compounds such as the CellMask™ dyes (Life Technologies). More recently, the MemBright dyes were developed allowing for higher photo-stability, lower working concentration and super-resolution microscopy applications (127). Alternatively, specific labels can be used to mimic the structure of a lipid or membrane constituent. This can be a lipid modified with an organic dye, a protein domain specifically binding to a lipid or even an antibody (126, 128). Labeled lipid analogs have enabled insightful studies of the dynamic nano-scale organization of the membrane (76, 77, 87). Nevertheless, due to the comparable size of dye and lipid it cannot be excluded that the analog does not exactly represent the native lipid. An important constituent of the plasma membrane with an abundance of about 30%–40% is cholesterol (35, 46). A variety of probes and cholesterol binding proteins are available (47, 129), however how cholesterol is distributed laterally and axially in the plasma membrane remains under heated debate.

It should be noted that fluorescent WGA conjugates are commonly used to stain the plasma membrane. This protein displays a lectin and rather binds the membrane adjacent glycocalyx thus spatial variations can occur within one cell and definitely when comparing different cell types (127). Some proteins have specific domains to interact with lipids. Such proteins can be tagged with a fluorescent protein and in this way be engineered to become a lipid reporter. These are convenient probes as the cells do not require any further labeling, but overexpression of such reporters may sequester the native lipid species, infer with endogenous signaling and membrane binding and lipid species specificity is often modulated by multiple components. Lipid binding domains include C1-domain (DAG), C2-domain (phospholipids), FYVE- and PX-domain (PI3P), PH-domain (phosphoinositide polyphosphates), and Annexin V (PS) (128, 130, 131).

A variety of compounds exists that not only label the membrane but also report on its properties (environment-sensitive dyes), known as functional probes. Polarity-sensitive dyes, for example, change their fluorescence properties depending on the local order (molecular packing, accessibility of the hydrophobic core to water from the exterior medium) of the surrounding membrane (132, 133). This results in quantifiable changes in fluorescence spectra or lifetimes and can be combined with super-resolution microscopy (134, 135). Other probes sense different membrane properties such as tension or viscosity (136, 137). Work with probes that change their spectrum upon changes in the local environment is experimentally convenient as it only requires acquisition in two optimized spectral channels (even though spectral detection is preferred) (138). Unfortunately, these dyes typically have a very broad emission spectrum that makes it difficult to simultaneously quantitatively image another structure such as actin in addition. Therefore, we anticipate again that lifetime-based probes and acquisitions may show better sensitivity (139, 140).

## Approaches for Quantifying F- and G-Actin Dynamics

Visualizing the dynamics of the actin cytoskeleton in living cells remains challenging, largely owing to the rapid turn-over of the molecular components of the cytoskeleton. Two main strategies exists for the visualization of actin within living cells: either genetic approaches modifying G-actin monomers directly with a fluorescent protein or a self-labeling tag (SNAP/Halo), or by using a variety of indirect filamentous F-actin binding labels, for example, the short peptides Lifeact or F-tractin, which are additionally modified with a fluorescent protein (141–143). As with lipid labeling, care must be taken to use the appropriate strategy for the desired dynamic output, for instance, FRAP or FCS studies assessing actin diffusion must utilize a direct G-actin approach to ensure that the measured diffusion is that of G-actin and not the indirect actin probe e.g. Lifeact-filament binding. A recent versatile approach is the visualization of membrane proximal F-actin by a membrane-bound F-tractin reporter (MPAct) as this directly reports on plasma membrane cortex interactions (144). This enables to investigate actin cortex remodeling in proximity of the membrane, which could yield novel insights into actin-rich protrusions and their dynamic reorganization during T-cell activation. Labeling F-actin in live cells can also be performed with a small organic molecule, SiR-actin (143). This is derived from a toxin and has the advantage that it can simply be added to the cell of interest, is cell permeable and can readily be used with super-resolution STED microscopy (145). Because of its mode of binding to actin filaments, it may perturb native actin dynamics. A rather recent approach displays the use of anti-actin nanobodies (Chromotek) (146–148) or direct delivery of mRNA encoding for labeled Lifeact with certain advantages for primary cells (IBIDI). In immunology both Lifeact and F-tractin have been widely applied to visualize F-actin dynamics (141, 149). More recently, Lifeact has also been implicated in changing endogenous actin dynamics (150–152). As with any secondary labeling, careful optimisation and controls are necessary. When investigating F-actin a comparison with a (fixed) phalloidin stain can serve as a control.

Common to all labeling strategies applied to fluorescence microscopy in living cells should be the use of probes that do not disturb the native behavior of the tagged molecule and do not influence the biological system. As mentioned above, lipids and the attached fluorescent dyes are of a comparable size, and thus measures should be taken to ensure that the lipid behaves as its native counterpart. Often a variety of chemical structures need to be screened in order to optimize labeling of a target molecule (i.e. signaling function, cellular localisation, molecular interactions and so forth need to be preserved and checked) (77, 129). Similar issues arise with the use of fluorescent proteins which can sterically hinder their target protein or artificially cause oligomerisation (153). Therefore, it is advisable to use flexible linkers and trial multiple labeling strategies, for example, C- and N-terminal tagging (154, 155). Overall, we would like to emphasize that controls ensuring the preserved function are of the utmost importance.

## Perturbation Studies Using Biochemical- and Photo-Manipulation

Specific labeling of molecules allows the membrane or the cytoskeleton to be studied in different physiological settings. A common additional step of the analysis is to perform perturbations, disturbing the steady-state of the actin assembly or membrane structure or even the actin-membrane interplay. By systematically perturbing different components of the system independently, the impact of each on the signaling and function within the living cell can be inferred and key-components, for example, as drug targets be identified.

Perturbations at the plasma membrane are commonly performed by altering the lipid composition. For transient changes, lipid species can simply be fed but recent evidence shows that the cells quickly counteract this to preserve the biophysical properties of the membrane (156). More commonly, certain membrane constituents are depleted. For instance, cholesterol which is a major component of the plasma membrane, playing an important role in signaling, and is proposed to be the major organizer of nanodomains aka rafts and has a profound effect on the overall biophysical properties such as viscosity and rigidity of the plasma membrane is a common target (46, 157). Treatments with cholesterol oxidase or methyl-β-cyclodextrin are routinely used to deplete cholesterol directly at the plasma membrane (158) for studying fast time scales such as seconds to minutes. Alternatively, drugs such as statins which interfere with cholesterol synthesis in the cell enable investigations of longer time scales up to hours and days (35). In that way, changes in cellular function, membrane organization or signaling upon variations in cholesterol content can be probed (77, 159). Analogously the role of sphingomyelin can be studied by treating the cell with sphingomyelinase or drugs such as fumonisin B1 or myriocin, respectively (35, 47, 77, 160).

Similarly, the actin cytoskeleton and its turnover can be targeted with various drugs or genetically modified (161, 162). For instance, Latruncullin B along with Cytochlasins or Phalloidin can be used to rapidly block F-actin polymerization (163), specifically inhibiting the addition of new actin monomers to the barbed end of F-actin (27, 28, 163). In contrast, Jasplakinolide can be used to stabilize actin filaments and promote polymerization (2, 164). Other drugs can be used to tune the cortex structure by influencing the nucleation of F-actin. For example, CK666 can inhibit actin branching by binding to Arp2/3 which can in turn be used to study the influence of the cortex organization and ultra-structure on the nano-scale diffusion behavior within the membrane (16, 165, 166). Furthermore, the formin inhibitor SMIFH2 can be used to remodel actin filaments and cortex structure (167, 168). In addition, a number of drugs can be used to target myosin molecular motors that drive stress generation within cortex. Specifically, both blebbistatin (169), an inhibitor of myosin II ATPase activity, and the rho kinase inhibitor Y27632 (170), are commonly used to perturb the ability of myosin II to actively generate stress within the cortex.

While these studies provided profound insights, for example, into actin reorganization during T-cell activation (27, 168), such perturbations using small molecules and enzymes affect the cell as a whole and can have unwanted side effects: For example, SMIFH2 has recently been indicated to inhibit myosins in addition to formins (171). Because of this, care needs to be taken when interpreting their effect on the structure or process of interest. A remedy to looking at global changes may come with the introduction of more photo-caged compounds or photo-activatable proteins, offering the potential for spatio-temporal control of perturbations (172–174). In addition, model systems such as cell-derived vesicles offer more control but do not allow to measure a living system (17, 35, 175).

## Discussion and Future Perspectives

The communication of immune cells with their environment, other immune cells and target cells involves a diversity of complex receptor-ligand interactions. These interactions all take place within the context of the plasma membrane and the underlying actin cortex. Consequently, their dynamics are intimately linked to the biophysical properties of both the membrane and the actin cytoskeleton and are constantly influencing one another. In this review, we have sought to highlight tools and technologies that present exciting opportunities to uncover the correlated dynamics of the plasma membrane and the cortical actin cytoskeleton at the immune cell interface for the first time, harnessing the power of simultaneous acquisitions. In particular, the presented methodologies provide a route to pick apart the key determinants of actin-membrane dynamics, unraveling the causal mechanistic relationships between the two systems. Crucially, these technologies operate across a wide range of length- and time-scales, allowing the investigation of nanoscale interactions on the sub-millisecond time scale up to large scale whole-cell measurements using volumetric imaging. Armed with these new technologies, immunologists can address key questions regarding the interactions of molecules on or in the proximity of the plasma membrane. The ubiquity of membrane interactions in immune cell functions means these techniques have the potential to provide insight into a wide range of immunological cell types, in both the adaptive and innate immune response.

As detailed, these new technologies together with novel functional probes allow the assessment of important biophysical parameters such as lipid order, charge, viscosity, and membrane tension. Many of these parameters have been implicated in immune cell function, thus their systematic probing using these newly available tools is timely (176–178). Coupled with established immunological methods, such as fluorescence-activated cell sorting (FACS), these techniques provide a powerful route to better understand the function of a wide range of immune cells.

To maximize the gains from the presented techniques, they must be applied at the appropriate time- and length-scales. FCS, for instance, operates at the μs to ms time scales and sub-micron spatial-scales, and FRAP rather on ms to s and on micron scales. MIET offers high sensitivity in the axial direction, but remains diffraction limited laterally, and temporal resolution is limited by the sample signal and lifetime acquisition (~s). Volumetric imaging can be extremely rapid, but in most cases cannot surpass the diffraction limit in spatial resolution. 3D-SIM is promising in the regard of isotropic sub-diffraction resolution but sacrifices temporal resolution (179, 180). Therefore, care needs to be taken to answer the right questions with the right tools. In addition to this, as discussed, probes and labels should be chosen such that they maximize the potential of the applied technique, for example, in FCS, dyes with a high molecular brightness and high photo-stability are desirable, whereas dyes utilized for FRAP should allow for efficient photobleaching. For MIET, fluorescent dyes showing a single exponential lifetime decay curve are desirable to allow for more straightforward reconstruction of the topological features from the quenched lifetimes. In addition, the labeling density should be considered: SPT, for example, can only be applied in the case that single molecules (single emitters) can be tracked, whereas FRAP and FCS can operate over a much wider range of molecular densities. Lastly, as for any multi-color microscopy experiments, the emission spectra and the possible overlap of the utilized dyes should be taken into account. Especially for dynamic techniques spectral bleed-through can result in the measurement of false positive interactions.

While these techniques present exciting opportunities for single cells and subcellular context, future work should focus on extending the capabilities of these methods to operate in more complex, more relevant multi-cellular environments including tissues and living organisms. Indeed, work in this direction is well underway with the introduction of rapid volumetric imaging systems like those presented here. We believe that great potential lies in the combination and integration of large scale volumetric imaging with technologies such as FC(C)S, FRAP and SPT, providing quantification of key biophysical parameters throughout the functionally diverse life cycles of immune cells.

## Author Contributions

FS, HC-Y, and MF wrote the manuscript. All authors contributed to the article and approved the submitted version.

## Conflict of Interest

The authors declare that the research was conducted in the absence of any commercial or financial relationships that could be construed as a potential conflict of interest.

## References

[1] Kumari S Curado S Mayya V Dustin ML T cell antigen receptor activation and actin cytoskeleton remodeling. Biochim Biophys Acta - Biomembr (2014) 1838:546–56. 10.1016/j.bbamem.2013.05.004 PMC3877165 23680625

[2] Colin-York H Javanmardi Y Skamrahl M Kumari S Chang VT Khuon S Cytoskeletal Control of Antigen-Dependent T Cell Activation. Cell Rep (2019) 26:3369–79.e5. 10.1016/j.celrep.2019.02.074 30893608 PMC6426652

[3] Mattila PK Batista FD Treanor B Dynamics of the actin cytoskeleton mediates receptor cross talk: An emerging concept in tuning receptor signaling. J Cell Biol (2016) 212:267–80. 10.1083/jcb.201504137 PMC4748574 26833785

[4] Gawden-Bone C Griffiths GM Phospholipids: Pulling back the actin curtain for granule delivery to the immune synapse. Front Immunol (2019) 10:700. 10.3389/fimmu.2019.00700 31031745 PMC6470250

[5] Köster DV Mayor S Cortical actin and the plasma membrane: inextricably intertwined. Curr Opin Cell Biol (2016) 38:81–9. 10.1016/j.ceb.2016.02.021 26986983

[6] Saarikangas J Zhao H Lappalainen P Regulation of the actin cytoskeleton-plasma membrane interplay by phosphoinositides. Physiol Rev (2010) 90:259–89. 10.1152/physrev.00036.2009 20086078

[7] Rottner K Faix J Bogdan S Linder S Kerkhoff E Actin assembly mechanisms at a glance. J Cell Sci (2017) 130:3427–35. 10.1242/jcs.206433 29032357

[8] Chugh P Paluch EK The actin cortex at a glance. J Cell Sci (2018) 131:jcs186254. 10.1242/jcs.186254 30026344 PMC6080608

[9] Fehon RG McClatchey AI Bretscher A Organizing the cell cortex: the role of ERM proteins. Nat Rev Mol Cell Biol (2010) 11:276–87. 10.1038/nrm2866 PMC2871950 20308985

[10] Mueller J Szep G Nemethova M de Vries I Lieber AD Winkler C Load Adaptation of Lamellipodial Actin Networks. Cell (2017) 171:188–200. 10.1016/j.cell.2017.07.051 28867286

[11] Schroer CFE Baldauf L van Buren L Wassenaar TA Melo MN Koenderink GH Charge-dependent interactions of monomeric and filamentous actin with lipid bilayers. Proc Natl Acad Sci U S A (2020) 117:5861–72. 10.1073/pnas.1914884117 PMC7084070 32123101

[12] Mogilner A Rubinstein B The physics of filopodial protrusion. Biophys J (2005) 89:782–95. 10.1529/biophysj.104.056515 PMC1366629 15879474

[13] Jung Y Riven I Feigelson SW Kartvelishvily E Tohya K Miyasaka M Three-dimensional localization of T-cell receptors in relation to microvilli using a combination of superresolution microscopies. Proc Natl Acad Sci U S A (2016) 113:E5916–24. 10.1073/pnas.1605399113 PMC5056101 27647916

[14] Schaumann EN Tian B Actin-packed topography: Cytoskeletal response to curvature. Proc Natl Acad Sci U S A (2019) 116:22897–8. 10.1073/pnas.1916656116 PMC6859312 31645378

[15] Wen PJ Grenklo S Arpino G Tan X Liao H-S Heureaux J Actin dynamics provides membrane tension to merge fusing vesicles into the plasma membrane. Nat Commun (2016) 7:12604. 10.1038/ncomms12604 27576662 PMC5013665

[16] Andrade DM Clausen MP Keller J Mueller V Wu C Bear JE Cortical actin networks induce spatio-temporal confinement of phospholipids in the plasma membrane – a minimally invasive investigation by STED-FCS. Sci Rep (2015) 5:11454. 10.1038/srep11454 26118385 PMC4484492

[17] Schneider F Waithe D Clausen MP Galiani S Koller T Ozhan G Diffusion of lipids and GPI-anchored proteins in actin-free plasma membrane vesicles measured by STED-FCS. Mol Biol Cell (2017) 28:1507–18. 10.1091/mbc.e16-07-0536 PMC5449149 28404749

[18] Simon C Caorsi V Campillo C Sykes C Interplay between membrane tension and the actin cytoskeleton determines shape changes. Phys Biol (2018) 15:065004. 10.1088/1478-3975/aad1ab 29978835

[19] Simon C Kusters R Caorsi V Allard A Abou-Ghali M Manzi J Actin dynamics drive cell-like membrane deformation. Nat Phys (2019) 8:602–9. 10.1038/s41567-019-0464-1

[20] Fooksman DR Vardhana S Vasiliver-Shamis G Liese J Blair DA Waite J Functional Anatomy of T Cell Activation and Synapse Formation. Annu Rev Immunol (2010) 28:79–105. 10.1146/annurev-immunol-030409-101308 19968559 PMC2885351

[21] Varma R Campi G Yokosuka T Saito T Dustin ML T Cell Receptor-Proximal Signals Are Sustained in Peripheral Microclusters and Terminated in the Central Supramolecular Activation Cluster. Immunity (2006) 25:117–27. 10.1016/j.immuni.2006.04.010 PMC1626533 16860761

[22] Bálint S Müller S Fischer R Kessler BM Harkiolaki M Valitutti S Supramolecular attack particles are autonomous killing entities released from cytotoxic T cells. Science (80- ) (2020) 368:897–901. 10.1126/science.aay9207 PMC7116847 32381591

[23] Cai E Marchuk K Beemiller P Beppler C Rubashkin MG Weaver VM Visualizing dynamic microvillar search and stabilization during ligand detection by T cells. Science (2017) 356:eaal3118. 10.1126/science.aal3118 28495700 PMC6364556

[24] Ghosh S Di Bartolo V Tubul L Shimoni E Kartvelishvily E Dadosh T ERM-Dependent Assembly of T Cell Receptor Signaling and Co-stimulatory Molecules on Microvilli prior to Activation. Cell Rep (2020) 30:3434–47. 10.1016/j.celrep.2020.02.069 32160548

[25] Jung Y Wen L Altman A Ley K CD45 Pre-Exclusion from the Tips of Microvilli Establishes a Phosphatase-Free Zone for Early TCR Triggering. BioRxiv (2020) 2020.05.21.109074. 10.1101/2020.05.21.109074

[26] Orbach R Su X Surfing on Membrane Waves: Microvilli, Curved Membranes, and Immune Signaling. Front Immunol (2020) 11:2187. 10.3389/fimmu.2020.02187 33013920 PMC7516127

[27] Fritzsche M Fernandes RA Chang VT Colin-York H Clausen MP Felce JH Cytoskeletal actin dynamics shape a ramifying actin network underpinning immunological synapse formation. Sci Adv (2017) 3:e1603032. 10.1126/sciadv.1603032 28691087 PMC5479650

[28] Yi J Wu XS Crites T Hammer JA Actin retrograde flow and actomyosin II arc contraction drive receptor cluster dynamics at the immunological synapse in Jurkat T cells. Mol Biol Cell (2012) 23:834–52. 10.1091/mbc.E11-08-0731 PMC3290643 22219382

[29] Babich A Li S O’Connor RS Milone MC Freedman BD Burkhardt JK. F-actin polymerization and retrograde flow drive sustained PLCγ1 signaling during T cell activation. J Cell Biol (2012) 197:775–87. 10.1083/jcb.201201018 PMC3373411 22665519

[30] Sims TN Soos TJ Xenias HS Dubin-Thaler B Hofman JM Waite JC Opposing Effects of PKCθ and WASp on Symmetry Breaking and Relocation of the Immunological Synapse. Cell (2007) 129:773–85. 10.1016/j.cell.2007.03.037 17512410

[31] Kumari S Mak M Poh Y Tohme M Watson N Melo M Cytoskeletal tension actively sustains the migratory T-cell synaptic contact. EMBO J (2020) 39:1–18. 10.15252/embj.2019102783 PMC7049817 31894880

[32] Gawden-Bone CM Frazer GL Richard AC Ma CY Strege K Griffiths GM PIP5 Kinases Regulate Membrane Phosphoinositide and Actin Composition for Targeted Granule Secretion by Cytotoxic Lymphocytes. Immunity (2018) 49:427–37. 10.1016/j.immuni.2018.08.017 PMC6162341 30217409

[33] Ritter AT Asano Y Stinchcombe JC Dieckmann NMG Chen BC Gawden-Bone C Actin Depletion Initiates Events Leading to Granule Secretion at the Immunological Synapse. Immunity (2015) 42:864–76. 10.1016/j.immuni.2015.04.013 PMC4448150 25992860

[34] Lorent JH Levental KR Ganesan L Rivera-Longsworth G Sezgin E Doktorova M Plasma membranes are asymmetric in lipid unsaturation, packing and protein shape. Nat Chem Biol (2020) 16:644–52. 10.1038/s41589-020-0529-6 PMC7246138 32367017

[35] Sezgin E Levental I Mayor S Eggeling C The mystery of membrane organization: composition, regulation and roles of lipid rafts. Nat Rev Mol Cell Biol (2017) 18:361–74. 10.1038/nrm.2017.16 PMC5500228 28356571

[36] Dinic J Riehl A Adler J Parmryd I The T cell receptor resides in ordered plasma membrane nanodomains that aggregate upon patching of the receptor. Sci Rep (2015) 5:10082. 10.1038/srep10082 25955440 PMC5386217

[37] Klammt C Lillemeier BF How membrane structures control T cell signaling. Front Immunol (2012) 3:291. 10.3389/fimmu.2012.00291 23055999 PMC3458435

[38] Meiri KF Lipid rafts and regulation of the cytoskeleton during T cell activation. Philos Trans R Soc B Biol Sci (2005) 360:1663–72. 10.1098/rstb.2005.1704 PMC1569545 16147530

[39] Montixi C Langlet C Bernard AM Thimonier J Dubois C Wurbel MA Engagement of T cell receptor triggers its recruitment to low-density detergent-insoluble membrane domains. EMBO J (1998) 17:5334–48. 10.1093/emboj/17.18.5334 PMC1170860 9736612

[40] Drevot P Langlet C Guo XJ Bernard AM Colard O Chauvin JP TCR signal initiation machinery is pre-assembled and activated in a subset of membrane rafts. EMBO J (2002) 21:1899–908. 10.1093/emboj/21.8.1899 PMC125369 11953309

[41] Gaus K Chklovskaia E Fazekas De St. Groth B Jessup W Harder T Condensation of the plasma membrane at the site of T lymphocyte activation. J Cell Biol (2005) 171:121–31. 10.1083/jcb.200505047 PMC2171224 16203859

[42] Lorent JH Diaz-Rohrer B Lin X Spring K Gorfe AA Levental KR Structural determinants and functional consequences of protein affinity for membrane rafts. Nat Commun (2017) 8:1219. 10.1038/s41467-017-01328-3 29089556 PMC5663905

[43] Sezgin E Levental I Grzybek M Schwarzmann G Mueller V Honigmann A Partitioning, diffusion, and ligand binding of raft lipid analogs in model and cellular plasma membranes. Biochim Biophys Acta (2012) 1818:1777–84. 10.1016/j.bbamem.2012.03.007 22450237

[44] Jacobson K Mouritsen OG Anderson RGW Lipid rafts: at a crossroad between cell biology and physics. Nat Cell Biol (2007) 9:7–14. 10.1038/ncb0107-7 17199125

[45] Ventimiglia LN Alonso MA The role of membrane rafts in Lck transport, regulation and signalling in T-cells. Biochem J (2013) 454:169–79. 10.1042/bj20130468 23931554

[46] Pinkwart K Schneider F Lukoseviciute M Sauka-Spengler T Lyman E Eggeling C Nanoscale dynamics of cholesterol in the cell membrane. J Biol Chem (2019) 294:12599–609. 10.1074/jbc.RA119.009683 PMC6709632 31270209

[47] Endapally S Frias D Grzemska M Gay A Tomchick DR Radhakrishnan A Molecular Discrimination between Two Conformations of Sphingomyelin in Plasma Membranes. Cell (2019) 176:1040–53. 10.1016/j.cell.2018.12.042 PMC6428426 30712872

[48] Subczynski WK Pasenkiewicz-Gierula M Widomska J Mainali L Raguz M High Cholesterol/Low Cholesterol: Effects in Biological Membranes Review. Cell Biochem Biophys (2017) 75:1–17. 10.1007/s12013-017-0792-7 28417231 PMC5645210

[49] Dinic J Ashrafzadeh P Parmryd I Actin filaments attachment at the plasma membrane in live cells cause the formation of ordered lipid domains. Biochim Biophys Acta - Biomembr (2013) 1828:1102–11. 10.1016/j.bbamem.2012.12.004 23246974

[50] Honigmann A Sadeghi S Keller J Hell SW Eggeling C Vink R A lipid bound actin meshwork organizes liquid phase separation in model membranes. Elife (2014) 2014:1–16. 10.7554/eLife.01671 PMC3957580 24642407

[51] Zumerle S Molon B Viola A Membrane rafts in T cell activation: A spotlight on CD28 costimulation. Front Immunol (2017) 8:1467. 10.3389/fimmu.2017.01467 29163534 PMC5675840

[52] Wickramarachchi DC Theofilopoulos AN Kono DH Immune pathology associated with altered actin cytoskeleton regulation. Autoimmunity (2010) 43:64–75. 10.3109/08916930903374634 20001423 PMC3660107

[53] Beemiller P Krummel MF Mediation of T-cell activation by actin meshworks. Cold Spring Harb Perspect Biol (2010) 2:1–14. 10.1101/cshperspect.a002444 PMC2926748 20702599

[54] Joosten B Willemse M Fransen J Cambi A van den Dries K Super-Resolution Correlative Light and Electron Microscopy (SR-CLEM) Reveals Novel Ultrastructural Insights Into Dendritic Cell Podosomes. Front Immunol (2018) 9:1908. 10.3389/fimmu.2018.01908 30186284 PMC6113363

[55] Dobbie IM Bridging the resolution gap: correlative super-resolution imaging. Nat Rev Microbiol (2019) 17:337. 10.1038/s41579-019-0203-8 31040390

[56] Ganeva I Kukulski W Membrane Architecture in the Spotlight of Correlative Microscopy. Trends Cell Biol (2020) 30:577–87. 10.1016/j.tcb.2020.04.003 32402740

[57] Ojcius DM Niedergang F Subtil A Hellio R Dautry-Varsat A Immunology and the confocal microscope. Res Immunol (1996) 147:175–88. 10.1016/0923-2494(96)83169-5 8817746

[58] Lee KH Holdorf AD Dustin ML Chan AC Allen PM Shaw AS T cell receptor signaling precedes immunological synapse formation. Science (80- ) (2002) 295:1539–42. 10.1126/science.1067710 11859198

[59] Harwood NE Batista FD Early Events in B Cell Activation. Annu Rev Immunol (2010) 28:185–210. 10.1146/annurev-immunol-030409-101216 20192804

[60] Beemiller P Krummel MF Regulation of T-cell receptor signaling by the actin cytoskeleton and poroelastic cytoplasm. Immunol Rev (2013) 256:148–59. 10.1111/imr.12120 PMC3831008 24117819

[61] Roy NH Burkhardt JK The actin cytoskeleton: A mechanical intermediate for signal integration at the immunological synapse. Front Cell Dev Biol (2018) 6:116. 10.3389/fcell.2018.00116 30283780 PMC6156151

[62] Lackowicz J Principles of Fluorescence Spectroscopy. 3rd ed. Lakowicz JR, editor. Boston, MA: Springer US (2006). 10.1007/978-0-387-46312-4

[63] Manzo C Garcia-Parajo MF A review of progress in single particle tracking: from methods to biophysical insights. Rep Prog Phys (2015) 78:124601. 10.1088/0034-4885/78/12/124601 26511974

[64] Dorsch S Klotz KN Engelhardt S Lohse MJ Bünemann M Analysis of receptor oligomerization by FRAP microscopy. Nat Methods (2009) 6:225–30. 10.1038/nmeth.1304 19234451

[65] Favier B Burroughs NJ Wedderburn L Valitutti S TCR dynamics on the surface of living T cells. Int Immunol (2001) 13:1525–32. 10.1093/intimm/13.12.1525 11717193

[66] Schwarzenbacher M Kaltenbrunner M Brameshuber M Hesch C Paster W Weghuber J Micropatterning for quantitative analysis of protein-protein interactions in living cells. Nat Methods (2008) 5:1053–60. 10.1038/nmeth.1268 18997782

[67] Tolentino TP Wu J Zarnitsyna VI Fang Y Dustin ML Zhu C Measuring diffusion and binding kinetics by contact area FRAP. Biophys J (2008) 95:920–30. 10.1529/biophysj.107.114447 PMC2440437 18390627

[68] Treanor B Depoil D Gonzalez-Granja A Barral P Weber M Dushek O The Membrane Skeleton Controls Diffusion Dynamics and Signaling through the B Cell Receptor. Immunity (2010) 32:187–99. 10.1016/j.immuni.2009.12.005 PMC2984614 20171124

[69] Zimmermann L Paster W Weghuber J Eckerstorfer P Stockinger H Schütz GJ Direct observation and quantitative analysis of Lck exchange between plasma membrane and cytosol in living T cells. J Biol Chem (2010) 285:6063–70. 10.1074/jbc.M109.025981 PMC2825400 20040600

[70] Hiramoto-Yamaki N Tanaka KAK Suzuki KGN Hirosawa KM Miyahara MSH Kalay Z Ultrafast Diffusion of a Fluorescent Cholesterol Analog in Compartmentalized Plasma Membranes. Traffic (2014) 15:583–612. 10.1111/tra.12163 24506328 PMC4265843

[71] Kapoor-Kaushik N Hinde E Compeer EB Yamamoto Y Kraus F Yang Z Distinct Mechanisms Regulate Lck Spatial Organization in Activated T Cells. Front Immunol (2016) 7:83. 10.3389/fimmu.2016.00083 27014263 PMC4782156

[72] Ma Y Benda A Nicovich PR Gaus K Measuring membrane association and protein diffusion within membranes with supercritical angle fluorescence microscopy. BioMed Opt Express (2016) 7:1561. 10.1364/BOE.7.001561 27446675 PMC4929661

[73] Ponjavic A McColl J Carr AR Santos AM Kulenkampff K Lippert A Single-Molecule Light-Sheet Imaging of Suspended T Cells. Biophys J (2018) 114:2200–11. 10.1016/j.bpj.2018.02.044 PMC5961759 29742413

[74] Bedard M Shrestha D Priestman DA Wang Y Schneider F Matute JD Sterile activation of invariant natural killer T cells by ER-stressed antigen-presenting cells. Proc Natl Acad Sci (2019) 116:23671–81. 10.1073/pnas.1910097116 PMC6876220 31690657

[75] Sezgin E Schneider F Galiani S Urbančič I Waithe D Lagerholm BC Measuring nanoscale diffusion dynamics in cellular membranes with super-resolution STED–FCS. Nat Protoc (2019) 14:1054–83. 10.1038/s41596-019-0127-9 30842616

[76] Eggeling C Ringemann C Medda R Schwarzmann G Sandhoff K Polyakova S Direct observation of the nanoscale dynamics of membrane lipids in a living cell. Nature (2009) 457:1159–62. 10.1038/nature07596 19098897

[77] Mueller V Ringemann C Honigmann A Schwarzmann G Medda R Leutenegger M STED Nanoscopy Reveals Molecular Details of Cholesterol- and Cytoskeleton-Modulated Lipid Interactions in Living Cells. Biophys J (2011) 101:1651–60. 10.1016/j.bpj.2011.09.006 PMC3183802 21961591

[78] Ruan Q Cheng MA Levi M Gratton E Mantulin WW Spatial-temporal studies of membrane dynamics: scanning fluorescence correlation spectroscopy (SFCS). Biophys J (2004) 87:1260–7. 10.1529/biophysj.103.036483 PMC1304464 15298928

[79] Waithe D Schneider F Chojnacki J Clausen MP Shrestha D de la Serna JB Optimized processing and analysis of conventional confocal microscopy generated scanning FCS data. Methods (2017) 140–141:62–73. 10.1016/j.ymeth.2017.09.010 PMC6026296 28963070

[80] Schneider F Waithe D Lagerholm BC Shrestha D Sezgin E Eggeling C Statistical Analysis of Scanning Fluorescence Correlation Spectroscopy Data Differentiates Free from Hindered Diffusion. ACS Nano (2018) 12:8540–6. 10.1021/acsnano.8b04080 PMC6117752 30028588

[81] Blouin CM Hamon Y Gonnord P Boularan C Kagan J Viaris de Lesegno C Glycosylation-Dependent IFN-γR Partitioning in Lipid and Actin Nanodomains Is Critical for JAK Activation. Cell (2016) 166:920–34. 10.1016/j.cell.2016.07.003 27499022

[82] Guzmán C Šolman M Ligabue A Blaževitš O Andrade DM Reymond L The efficacy of raf kinase recruitment to the GTPase H-ras depends on H-ras membrane conformer-specific nanoclustering. J Biol Chem (2014) 289:9519–33. 10.1074/jbc.M113.537001 PMC3975003 24569991

[83] Ries J Yu SR Burkhardt M Brand M Schwille P Modular scanning FCS quantifies receptor-ligand interactions in living multicellular organisms. Nat Methods (2009) 6:643–5. 10.1038/nmeth.1355 19648917

[84] Di Bona M Mancini MA Mazza D Vicidomini G Diaspro A Lanzanò L Measuring Mobility in Chromatin by Intensity-Sorted FCS. Biophys J (2019) 116:987–99. 10.1016/j.bpj.2019.02.003 PMC6428914 30819566

[85] Dörlich RM Chen Q Niklas Hedde P Schuster V Hippler M Wesslowski J Dual-color dual-focus line-scanning FCS for quantitative analysis of receptor-ligand interactions in living specimens. Sci Rep (2015) 5:10149. 10.1038/srep10149 25951521 PMC4423563

[86] Dunsing V Mayer M Liebsch F Multhaup G Chiantia S Direct evidence of amyloid precursor–like protein 1 trans interactions in cell–cell adhesion platforms investigated via fluorescence fluctuation spectroscopy. Mol Biol Cell (2017) 28:3609–20. 10.1091/mbc.E17-07-0459 PMC5706989 29021345

[87] Honigmann A Mueller V Ta H Schoenle A Sezgin E Hell SW Scanning STED-FCS reveals spatiotemporal heterogeneity of lipid interaction in the plasma membrane of living cells. Nat Commun (2014) 5:5412. 10.1038/ncomms6412 25410140

[88] Schneider F Waithe D Galiani S Bernardino de la Serna J Sezgin E Eggeling C Nanoscale Spatiotemporal Diffusion Modes Measured by Simultaneous Confocal and Stimulated Emission Depletion Nanoscopy Imaging. Nano Lett (2018) 18:4233–40. 10.1021/acs.nanolett.8b01190 PMC6047073 29893574

[89] Lanzanò L Scipioni L Di Bona M Bianchini P Bizzarri R Cardarelli F Measurement of nanoscale three-dimensional diffusion in the interior of living cells by STED-FCS. Nat Commun (2017) 8:65. 10.1038/s41467-017-00117-2 28684735 PMC5500520

[90] Schneider F Hernandez-Varas P Christoffer Lagerholm B Shrestha D Sezgin E Julia Roberti M High photon count rates improve the quality of super-resolution fluorescence fluctuation spectroscopy. J Phys D Appl Phys (2020) 53:164003. 10.1088/1361-6463/ab6cca 33191951 PMC7655148

[91] Bag N Holowka DA Baird BA Imaging FCS delineates subtle heterogeneity in plasma membranes of resting mast cells. Mol Biol Cell (2020) 31:709–23. 10.1091/mbc.E19-10-0559 PMC7202073 31895009

[92] Wohland T Shi X Sankaran J Stelzer EHK Single Plane Illumination Fluorescence Correlation Spectroscopy (SPIM-FCS) probes inhomogeneous three-dimensional environments. Opt Express (2010) 18:10627. 10.1364/oe.18.010627 20588915

[93] Bag N Sankaran J Paul A Kraut RS Wohland T Calibration and limits of camera-based fluorescence correlation spectroscopy: A supported lipid bilayer study. ChemPhysChem (2012) 13:2784–94. 10.1002/cphc.201200032 22615144

[94] Di Rienzo C Gratton E Beltram F Cardarelli F Fast spatiotemporal correlation spectroscopy to determine protein lateral diffusion laws in live cell membranes. Proc Natl Acad Sci (2013) 110:12307–12. 10.1073/pnas.1222097110 PMC3725058 23836651

[95] Di Rienzo C Gratton E Beltram F Cardarelli F Spatiotemporal Fluctuation Analysis: A Powerful Tool for the Future Nanoscopy of Molecular Processes. Biophys J (2016) 111:679–85. 10.1016/j.bpj.2016.07.015 PMC5002078 27558712

[96] Di Rienzo C Gratton E Beltram F Cardarelli F From Fast Fluorescence Imaging to Molecular Diffusion Law on Live Cell Membranes in a Commercial Microscope. J Vis Exp (2014) e51994:1–12. 10.3791/51994 PMC4461222 25350683

[97] Ortega-Arroyo J Kukura P Interferometric scattering microscopy (iSCAT): new frontiers in ultrafast and ultrasensitive optical microscopy. Phys Chem Chem Phys (2012) 14:15625. 10.1039/c2cp41013c 22996289

[98] Reina F Galiani S Shrestha D Sezgin E De Wit G Cole D Complementary studies of lipid membrane dynamics using iSCAT and super-resolved fluorescence correlation spectroscopy. J Phys D Appl Phys (2018) 51:235401. 10.1088/1361-6463/aac04f 29853718 PMC5964363

[99] Taylor RW Mahmoodabadi RG Rauschenberger V Giessl A Schambony A Sandoghdar V Interferometric scattering microscopy reveals microsecond nanoscopic protein motion on a live cell membrane. Nat Photonics (2019) 13:480–7. 10.1038/s41566-019-0414-6

[100] Alvarez LAJ Widzgowski B Ossato G van den BB Jalink K Kuschel L SP8 FALCON : a novel concept in fluorescence lifetime imaging enabling video-rate confocal FLIM. Nat Methods (2019). 10.1038/d42473-019-00261

[101] Sun Y Day RN Periasamy A Investigating protein-protein interactions in living cells using fluorescence lifetime imaging microscopy. Nat Protoc (2011) 6:1324–40. 10.1038/nprot.2011.364 PMC3169422 21886099

[102] Chizhik AI Rother J Gregor I Janshoff A Enderlein J Metal-induced energy transfer for live cell nanoscopy. Nat Photonics (2014) 8:124–7. 10.1038/nphoton.2013.345

[103] Benda A Fagul’ová V Deyneka A Enderlein J Hof M Fluorescence lifetime correlation spectroscopy combined with lifetime tuning: New perspectives in supported phospholipid bilayer research. Langmuir (2006) 22:9580–5. 10.1021/la061573d 17073482

[104] Kułakowska A Jurkiewicz P Sýkora J Benda A Mely Y Hof M Fluorescence lifetime tuning-a novel approach to study flip-flop kinetics in supported phospholipid bilayers. J Fluoresc (2010) 20:563–9. 10.1007/s10895-009-0581-9 20039107

[105] Ghosh A Sharma A Chizhik AI Isbaner S Ruhlandt D Tsukanov R Graphene-based metal-induced energy transfer for sub-nanometre optical localization. Nat Photonics (2019) 13:860–5. 10.1038/s41566-019-0510-7

[106] Jarsch IK Daste F Gallop JL Membrane curvature in cell biology: An integration of molecular mechanisms. J Cell Biol (2016) 214:375–87. 10.1083/jcb.201604003 PMC4987295 27528656

[107] Baronsky T Ruhlandt D Brueckner BR Schäfer J Karedla N Haehnel D Cell-Substrate Dynamics of the Epithelial-to- Mesenchymal Transition. Nano Lett (2017) 17(5):3320–6. 10.1021/acs.nanolett.7b01558 28440076

[108] Chizhik AM Wollnik C Ruhlandt D Karedla N Chizhik AI Hauke L Dual-color metal-induced and förster resonance energy transfer for cell nanoscopy. Mol Biol Cell (2018) 29:846–51. 10.1091/mbc.E17-05-0314. mbc.E17-05-0314. PMC5905297 29444956

[109] Chizhik AM Ruhlandt D Pfaff J Karedla N Chizhik AI Gregor I Three Dimensional Reconstruction of Nuclear Envelope Architecture Using Dual-Color Metal-Induced Energy Transfer Imaging. ACS Nano (2017) 11:11839–46. 10.1021/acsnano.7b04671. acsnano.7b04671. 28921961

[110] Ghosh A Karedla N Thiele JC Gregor I Enderlein J Fluorescence lifetime correlation spectroscopy: Basics and applications. Methods (2018) 140–141:32–9. 10.1016/j.ymeth.2018.02.009 29454862

[111] Chang BJ Kittisopikul M Dean KM Roudot P Welf ES Fiolka R Universal light-sheet generation with field synthesis. Nat Methods (2019) 16(3):235–8. 10.1038/s41592-019-0327-9 PMC6561754 30804550

[112] Millett-Sikking A York A AndrewGYork/high_na_single_objective_lightsheet: Work-in-progress (Version 0.0.1). Zenodo (2019). 10.5281/zenodo.3244421

[113] Yang B Millett-Sikking A Lange M Solak AC Kobayashi H York A High-Resolution , Large Field-of-View , and Multi-View Single Objective Light-Sheet Microscopy. bioRxiv (2020), 1–10. 10.1101/2020.09.22.309229

[114] McDole K Guignard L Amat F Berger A Malandain G Royer LA In Toto Imaging and Reconstruction of Post-Implantation Mouse Development at the Single-Cell Level. Cell (2018) 175:859–76.e33. 10.1016/j.cell.2018.09.031 30318151

[115] Voleti V Patel KB Li W Perez Campos C Bharadwaj S Yu H Real-time volumetric microscopy of in vivo dynamics and large-scale samples with SCAPE 2.0. Nat Methods (2019) 16:1054–62. 10.1038/s41592-019-0579-4 PMC6885017 31562489

[116] Chen BCB-C Legant WR Wang K Shao L Milkie DE Davidson MW Lattice light-sheet microscopy: Imaging molecules to embryos at high spatiotemporal resolution. Science (2014) 346:1257998–1257998. 10.1126/science.1257998 25342811 PMC4336192

[117] Groves JT Dustin ML Supported planar bilayers in studies on immune cell adhesion and communication. J Immunol Methods (2003) 278:19–32. 10.1016/S0022-1759(03)00193-5 12957393

[118] Santos AM Ponjavic A Fritzsche M Fernandes RA de la Serna JB Wilcock MJ Capturing resting T cells: the perils of PLL. Nat Immunol (2018) 19:203–5. 10.1038/s41590-018-0048-8 PMC7612954 29476188

[119] Jenkins E Santos AM O’Brien-Ball C Felce JH Wilcock MJ Hatherley D Reconstitution of immune cell interactions in free-standing membranes. J Cell Sci (2019) 132:jcs219709. 10.1242/jcs.219709 PMC6398472 30209137

[120] Roybal KT Mace EM Mantell JM Verkade P Orange JS Wülfing C Early signaling in primary t cells activated by antigen presenting cells is associated with a deep and transient lamellal actin network. PLoS One (2015) 10:1–22. 10.1371/journal.pone.0133299 PMC4523204 26237050

[121] Rosenberg J Cao G Borja-Prieto F Huang J Lattice Light-Sheet Microscopy Multi-dimensional Analyses (LaMDA) of T-Cell Receptor Dynamics Predict T-Cell Signaling States. Cell Syst (2020) 10:433–44. 10.1016/j.cels.2020.04.006 PMC7250142 32437685

[122] Fritzsche M Charras G Dissecting protein reaction dynamics in living cells by fluorescence recovery after photobleaching. Nat Protoc (2015) 10:660–80. 10.1038/nprot.2015.042 25837418

[123] Hobson CM O’Brien ET Falvo MR Superfine R Combined Selective Plane Illumination Microscopy and FRAP Maps Intranuclear Diffusion of NLS-GFP. Biophys J (2020) 119:514–24. 10.1016/j.bpj.2020.07.001 PMC7399492 32681822

[124] Sims PJ Waggoner AS Wang CH Hoffman JF Mechanism by which cyanine dyes measure membrane potential in red blood cells and phosphatidylcholine vesicles. Biochemistry (1974) 13:3315–30. 10.1021/bi00713a022 4842277

[125] Hume RI Honig MG Fluorescent Carbocyanine Dyes Allow Living Neurons of Identified Origin to Be Studied in Long-Term Cultures. J Cell Biol (1986) 103:171–87. 10.1083/jcb.103.1.171 PMC2113786 2424918

[126] Klymchenko AS Kreder R Fluorescent probes for lipid rafts: From model membranes to living cells. Chem Biol (2014) 21:97–113. 10.1016/j.chembiol.2013.11.009 24361047

[127] Collot M Ashokkumar P Anton H Boutant E Faklaris O Galli T MemBright: A Family of Fluorescent Membrane Probes for Advanced Cellular Imaging and Neuroscience. Cell Chem Biol (2019) 26:600–14. 10.1016/j.chembiol.2019.01.009 30745238

[128] Maekawa M Fairn GD Molecular probes to visualize the location, organization and dynamics of lipids. J Cell Sci (2014) 127:4801–12. 10.1242/jcs.150524 25179600

[129] Sezgin E Can FB Schneider F Clausen MP Galiani S Stanly TA A comparative study on fluorescent cholesterol analogs as versatile cellular reporters. J Lipid Res (2016) 57:299–309. 10.1194/jlr.M065326 26701325 PMC4727425

[130] Hurley JH Misra S Signaling and subcellular targeting by membrane-binding domains. Annu Rev Biophys Biomol Struct (2000) 29:49–79. 10.1146/annurev.biophys.29.1.49 10940243 PMC4781318

[131] Várnai P Gulyás G Tóth DJ Sohn M Sengupta N Balla T Quantifying lipid changes in various membrane compartments using lipid binding protein domains. Cell Calcium (2017) 64:72–82. 10.1016/j.ceca.2016.12.008 28088320 PMC5457350

[132] Parasassi T Krasnowska EK Laurdan and Prodan as polarity-sensitive fluorescent membrane probes. J Fluoresc (1998) 8:365–73. 10.1023/A:1020528716621

[133] Sezgin E Sadowski T Simons K Measuring lipid packing of model and cellular membranes with environment sensitive probes. Langmuir (2014) 30:8160–6. 10.1021/la501226v 24905799

[134] Sezgin E Schneider F Zilles V Urbančič I Garcia E Waithe D Polarity-Sensitive Probes for Superresolution Stimulated Emission Depletion Microscopy. Biophys J (2017) 113:1321–30. 10.1016/j.bpj.2017.06.050 PMC5607142 28734477

[135] Danylchuk DI Moon S Xu K Klymchenko AS Switchable Solvatochromic Probes for Live-Cell Super-resolution Imaging of Plasma Membrane Organization. Angew Chemie (2019) 131:15062–6. 10.1002/ange.201907690 31392763

[136] Colom A Derivery E Soleimanpour S Tomba C Molin MD Sakai N A fluorescent membrane tension probe. Nat Chem (2018) 10:1118–25. 10.1038/s41557-018-0127-3 PMC6197433 30150727

[137] Dal Molin M Verolet Q Colom A Letrun R Derivery E Gonzalez-Gaitan M Fluorescent Flippers for Mechanosensitive Membrane Probes. J Am Chem Soc (2015) 137:568–71. 10.1021/ja5107018 PMC4308758 25584496

[138] Sezgin E Waithe D Bernardino de la Serna J Eggeling C Spectral Imaging to Measure Heterogeneity in Membrane Lipid Packing. ChemPhysChem (2015) 16:1387–94. 10.1002/cphc.201402794 PMC4539592 25755090

[139] Steinkühler J Sezgin E Urbančič I Eggeling C Dimova R Mechanical properties of plasma membrane vesicles correlate with lipid order and viscosity and depend on cell density. Commun Biol (2019) 2:1–18. 10.1101/669085 31531398 PMC6744421

[140] Owen DM Lanigan PMP Dunsby C Munro I Grant D Neil MAA Fluorescence lifetime imaging provides enhanced contrast when imaging the phase-sensitive dye di-4-ANEPPDHQ in model membranes and live cells. Biophys J (2006) 90:L80–2. 10.1529/biophysj.106.084673 PMC1459501 16617080

[141] Riedl J Crevenna AH Kessenbrock K Yu JH Neukirchen D Bista M Lifeact: A versatile marker to visualize F-actin. Nat Methods (2008) 5:605–7. 10.1038/nmeth.1220 PMC2814344 18536722

[142] Johnson HW Schell MJ Neuronal IP 3 3-Kinase is an F-actin–bundling Protein: Role in Dendritic Targeting and Regulation of Spine Morphology. Mol Biol Cell (2009) 20:5166–80. 10.1091/mbc.e09-01-0083 PMC2793293 19846664

[143] Melak M Plessner M Grosse R Actin visualization at a glance. J Cell Sci (2017) 130:525–30. 10.1242/jcs.189068 28082420

[144] Bisaria A Hayer A Garbett D Cohen D Meyer T Membrane-proximal F-actin restricts local membrane protrusions and directs cell migration. Science (80- ) (2020) 368:1205–10. 10.1126/science.aay7794 PMC8283920 32527825

[145] D’Este E Kamin D Göttfert F El-Hady A Hell SW STED Nanoscopy Reveals the Ubiquity of Subcortical Cytoskeleton Periodicity in Living Neurons. Cell Rep (2015) 10:1246–51. 10.1016/j.celrep.2015.02.007 25732815

[146] Plessner M Melak M Chinchilla P Baarlink C Grosse R Nuclear F-actin formation and reorganization upon cell spreading. J Biol Chem (2015) 290:11209–16. 10.1074/jbc.M114.627166 PMC4416828 25759381

[147] Rocchetti A Hawes C Kriechbaumer V Fluorescent labelling of the actin cytoskeleton in plants using a cameloid antibody. Plant Methods (2014) 10:12. 10.1186/1746-4811-10-12 24872838 PMC4036722

[148] Schiavon C Zhang T Zhao B Andrade L Wu M Sung T-C Actin chromobody imaging reveals sub-organellar actin dynamics. Nat Methods (2019) 17:917–21. 10.1101/639278 PMC7746311 32778832

[149] Colin-York H Kumari S Barbieri L Cords L Fritzsche M Distinct actin cytoskeleton behaviour in primary and immortalised T-cells. J Cell Sci (2019) 133:jcs232322. 10.1242/jcs.232322 31413071 PMC6898998

[150] Courtemanche N Pollard TD Chen Q Avoiding artefacts when counting polymerized actin in live cells with LifeAct fused to fluorescent proteins. Nat Cell Biol (2016) 18:676–83. 10.1038/ncb3351 PMC5509211 27159499

[151] Spracklen AJ Fagan TN Lovander KE Tootle TL The pros and cons of common actin labeling tools for visualizing actin dynamics during Drosophila oogenesis. Dev Biol (2014) 393:209–26. 10.1016/j.ydbio.2014.06.022 PMC4438707 24995797

[152] Montes-Rodriguez A Kost B Direct comparison of the performance of commonly employed in vivo F-actin markers (Lifeact-YFP, YFP-mTn and YFP-FABD2) in tobacco pollen tubes. Front Plant Sci (2017) 8:1349. 10.3389/fpls.2017.01349 28824684 PMC5540898

[153] Ai HW Baird MA Shen Y Davidson MW Campbell RE Engineering and characterizing monomeric fluorescent proteins for live-cell imaging applications. Nat Protoc (2014) 9:910–28. 10.1038/nprot.2014.054 24651502

[154] Snapp E Design and Use of Fluorescent Fusion Proteins in Cell Biology. Curr Protoc Cell Biol (2005) 27:21.4.1–21.4.13. 10.1002/0471143030.cb2104s27 18228466 PMC2875081

[155] Ooi A Wong A Esau L Lemtiri-Chlieh F Gehring C. A Guide to Transient Expression of Membrane Proteins in HEK-293 Cells for Functional Characterization. Front Physiol (2016) 7:300. 10.3389/fphys.2016.00300 27486406 PMC4949579

[156] Levental KR Malmberg E Symons JL Fan YY Chapkin RS Ernst R Lipidomic and biophysical homeostasis of mammalian membranes counteracts dietary lipid perturbations to maintain cellular fitness. Nat Commun (2020) 11:1–13. 10.1038/s41467-020-15203-1 32165635 PMC7067841

[157] Lingwood D Simons K Lipid rafts as a membrane-organizing principle. Science (2010) 327:46–50. 10.1126/science.1174621 20044567

[158] Neuvonen M Manna M Mokkila S Javanainen M Rog T Liu Z Enzymatic oxidation of cholesterol: Properties and functional effects of cholestenone in cell membranes. PLoS One (2014) 9:e103743. 10.1371/journal.pone.0103743 25157633 PMC4144813

[159] Rouquette-Jazdanian AK Pelassy C Breittmayer JP Aussel C Revaluation of the role of cholesterol in stabilizing rafts implicated in T cell receptor signaling. Cell Signal (2006) 18:105–22. 10.1016/j.cellsig.2005.03.024 15925486

[160] Storti B Di Rienzo C Cardarelli F Bizzarri R Beltram F Unveiling TRPV1 spatio-temporal organization in live cell membranes. PLoS One (2015) 10:e0116900. 10.1371/journal.pone.0116900 25764349 PMC4357434

[161] Silvestre F Tosti E Impact of marine drugs on cytoskeleton-mediated reproductive events. Mar Drugs (2010) 8:881–915. 10.3390/md8040881 20479959 PMC2866467

[162] Harterink M Da Silva ME Will L Turan J Ibrahim A Lang AE DeActs: Genetically encoded tools for perturbing the actin cytoskeleton in single cells. Nat Methods (2017) 14:479–82. 10.1038/nmeth.4257 PMC5419720 28394337

[163] Cooper JA Effects of cytochalasin and phalloidin on actin. J Cell Biol (1987) 105:1473–8. 10.1083/jcb.105.4.1473 PMC2114638 3312229

[164] Holzinger A Jasplakinolide: An Actin-Specific Reagent that Promotes Actin Polymerization. In: Gavin RH, editor. Cytoskeleton Methods and Protocols. Totowa, NJ: Humana Press (2009). p. 71–87. 10.1007/978-1-60761-376-3_4 19768425

[165] Fritzsche M Erlenkämper C Moeendarbary E Charras G Kruse K Actin kinetics shapes cortical network structure and mechanics. Sci Adv (2016) 2:e1501337. 10.1126/sciadv.1501337 27152338 PMC4846455

[166] Fritzsche M Li D Colin-York H Chang VT Moeendarbary E Felce JH Self-organizing actin patterns shape membrane architecture but not cell mechanics. Nat Commun (2017) 8:14347. 10.1038/ncomms14347 28194011 PMC5316839

[167] Isogai T Van Der Kammen R Innocenti M SMIFH2 has effects on Formins and p53 that perturb the cell cytoskeleton. Sci Rep (2015) 5:9802. 10.1038/srep09802 25925024 PMC5386218

[168] Murugesan S Hong J Yi J Li D Beach JR Shao L Formin-generated actomyosin arcs propel t cell receptor microcluster movement at the immune synapse. J Cell Biol (2016) 215:383–99. 10.1083/jcb.201603080 PMC5100289 27799367

[169] Kovács M Tóth J Hetényi C Málnási-Csizmadia A Sellers JR Mechanism of Blebbistatin Inhibition of Myosin II. J Biol Chem (2004) 279:35557–63. 10.1074/jbc.M405319200 15205456

[170] Ishizaki T Uehata M Tamechika I Keel J Nonomura K Maekawa M Pharmacological properties of Y-27632, a specific inhibitor of rho-associated kinases. Mol Pharmacol (2000) 57:976–83. 10779382

[171] Sellers JR Shi S Nishimura Y Zhang F Liu R Takagi Y The Formin Inhibitor, SMIFH2, Inhibits Members of the Myosin Superfamily. Biophys J (2020) 118:125a. 10.1016/j.bpj.2019.11.817 PMC8121067 33589498

[172] Ross TD Lee HJ Qu Z Banks RA Phillips R Thomson M Controlling organization and forces in active matter through optically defined boundaries. Nature (2019) 572:224–9. 10.1038/s41586-019-1447-1 PMC6719720 31391558

[173] Schuhmacher M Grasskamp AT Barahtjan P Wagner N Lombardot B Schuhmacher JS Live-cell lipid biochemistry reveals a role of diacylglycerol side-chain composition for cellular lipid dynamics and protein affinities. Proc Natl Acad Sci U S A (2020) 117:7729–38. 10.1073/pnas.1912684117 PMC7149225 32213584

[174] Ballister ER Aonbangkhen C Mayo AM Lampson MA Chenoweth DM Localized light-induced protein dimerization in living cells using a photocaged dimerizer. Nat Commun (2014) 5:5475. 10.1038/ncomms6475 25400104 PMC4308733

[175] Sezgin E Kaiser H-J Baumgart T Schwille P Simons K Levental I Elucidating membrane structure and protein behavior using giant plasma membrane vesicles. Nat Protoc (2012) 7:1042–51. 10.1038/nprot.2012.059 22555243

[176] Rossy J Laufer JM Legler DF Role of mechanotransduction and tension in t cell function. Front Immunol (2018) 9:2638. 10.3389/fimmu.2018.02638 30519239 PMC6251326

[177] Rudd-Schmidt JA Hodel AW Noori T Lopez JA Cho H-J Verschoor S Lipid order and charge protect killer T cells from accidental death. Nat Commun (2019) 10:5396. 10.1038/s41467-019-13385-x 31776337 PMC6881447

[178] Limozin L Puech PH Membrane Organization and Physical Regulation of Lymphocyte Antigen Receptors: A Biophysicist’s Perspective. J Membr Biol (2019) 252:397–412. 10.1007/s00232-019-00085-2 31352492

[179] Wu Y Shroff H Faster, sharper, and deeper: structured illumination microscopy for biological imaging. Nat Methods (2018) 15:1011–9. 10.1038/s41592-018-0211-z 30478322

[180] Kraus F Miron E Demmerle J Chitiashvili T Budco A Alle Q Quantitative 3D structured illumination microscopy of nuclear structures. Nat Protoc (2017) 12:1011–28. 10.1038/nprot.2017.020 28406495

